# Withaferin A Exerts Preventive Effect on Liver Fibrosis through Oxidative Stress Inhibition in a Sirtuin 3-Dependent Manner

**DOI:** 10.1155/2020/2452848

**Published:** 2020-09-24

**Authors:** Jingya Gu, Chang Chen, Jue Wang, Tingting Chen, Wenjuan Yao, Tingdong Yan, Zhaoguo Liu

**Affiliations:** School of Pharmacy, Nantong University, 19 Qixiu Road, Nantong, Jiangsu Province 226001, China

## Abstract

Sirtuin 3 (SIRT3) is a deacetylase involved in the development of many inflammation-related diseases including liver fibrosis. Withaferin A (WFA) is a bioactive constituent derived from the *Withania somnifera* plant, which has extensive pharmacological activities; however, little is known about the regulatory role of SIRT3 in the WFA-induced antifibrogenic effect. The current study is aimed at investigating the role of SIRT3 in WFA-induced antioxidant effects in liver fibrosis. Our study verified that WFA attenuated platelet-derived growth factor BB- (PDGF-BB-) induced liver fibrosis and promoted PDGF-BB-induced SIRT3 activity and expression in JS1 cells. SIRT3 silencing attenuated the antifibrogenic and antioxidant effects of WFA in activated JS1 cells. Moreover, WFA inhibited carbon tetrachloride- (CCl_4_-) induced liver injury, collagen deposition, and fibrosis; increased the SIRT3 expression; and suppressed the CCl_4_-induced oxidative stress in fibrotic livers of C57/BL6 mice. Furthermore, the antifibrogenic and antioxidant effects of WFA could be available in CCl_4_-induced WT (129S1/SvImJ) mice but were unavailable in CCl_4_-induced SIRT3 knockout (KO) mice. Our study suggested that WFA inhibited liver fibrosis through the inhibition of oxidative stress in a SIRT3-dependent manner. WFA could be a potential compound for the treatment of liver fibrosis.

## 1. Introduction

Liver fibrosis is a reversible wound-healing response secondary to various chronic liver injuries during tissue repair, which is characterized by the excessive extracellular matrix (ECM) [1]. Liver fibrosis can be triggered by many risk factors, such as chemical damage, virus infection, alcohol abuse, and autoimmune disorders [2, 3]. Without effective treatment, the continuous progression of the disease will lead to the formation of liver fiber nodules and destroy the normal liver structure and function, and may eventually develop into cirrhosis or even liver cancer [4]. The activation of hepatic stellate cells (HSCs) by various stimuli, such as platelet-derived growth factor (PDGF) or transforming growth factor *β* (TGF-*β*), is considered as the key step of the development of liver fibrosis [5]. Upon activation, HSCs will undergo phenotypic transformation, acquire myofibroblast characteristics, and further increase collagen and ECM synthesis and cell contractility [6]. Therefore, HSCs are regarded as the main cellular target in the treatment of liver fibrosis.

Compelling evidence indicates that oxidative stress is involved in the development of liver fibrosis [7]. Oxidative stress is caused by the imbalance between the formation and removal of reactive oxygen species (ROS) in the liver, and it is considered to be the initial stage of liver fibrogenesis [8]. Sustained oxidative stress in the liver acts directly or indirectly on liver cells and changes the structure of the liver cell membrane and organelles, leading to damage, necrosis, apoptosis, and production of a large number of cytokines in liver cells [9, 10]. These cytokines will further activate the Kupffer cells and cause more cytokine secretion, which aggravate the liver injury [11]. The antioxidant system is a defense mechanism against oxidative stress in the liver [12]. Enhancement of the activities of some antioxidant enzymes such as catalase (CAT), glutathione reductase (GR), glutathione peroxidase (GPx), heme oxygenase 1 (HO-1), and superoxide dismutase (SOD) can inhibit oxidative stress and thereby delay the development of liver fibrosis [13]. Regulation of the antioxidant system has been considered as an effective strategy against liver fibrosis.

Silent information regulator 2 (SIR2) is a family of nicotinamide adenine dinucleotide- (NAD-) dependent histone deacetylases [14]. Mammals have seven different sirtuins, i.e., SIRT1-SIRT7, with different subcellular localizations and functions. SIRT1 and 6 are mainly located in the nucleus. SIRT3, 4, and 5 are mainly located in mitochondria [15]. SIRT2 is the only sirtuin that is mainly located in the cytoplasm. These proteins play important roles in critical cellular processes such as cell cycle control, maintenance of mitochondrial homeostasis, caloric restriction, and regulation of various glucose and lipid metabolism [16, 17]. Noteworthy, of all the seven sirtuins, SIRT3 is proven to be closely associated with oxidative stress [18]. SIRT3 depletion usually concomitant with the higher oxidation level and activation of SIRT3 helps the control of oxidative stress [19]. Moreover, SIRT3 knockout mice do not show any noticeable phenotype at birth; because of this reason, it is believed that SIRT3 does not play a role in embryonic development, but rather, it fine tunes the activity of mitochondrial substrates by lysine deacetylation to protect cells from stress [20]. Growing evidence indicates that SIRT3 is beneficial in the prevention of liver-related diseases including nonalcoholic fatty liver, liver injury, and fibrosis [21]. Studies found that SIRT3 deficiency aggravated the carbon tetrachloride- (CCl_4_-) induced liver injury [22], while activation of SIRT3 contributed to the attenuation of liver fibrosis [15].

**Figure 1:**
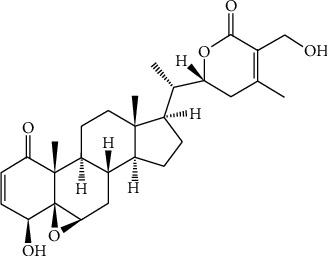
Chemical structure of withaferin A.

Studies have shown that herbal plant-derived active ingredients have unique advantages in the treatment of liver fibrosis [23]. In particular, these ingredients are widely distributed, readily available, and have fewer side effects [24]. Withaferin A (WFA) is a steroidal lactone (the chemical structure of WFA is shown in Figure 1) derived from the herbal plant *Withania somnifera* (L.) Dunal (Solanaceae), which has been used for the treatment of many diseases in Asia and Africa [25]. WFA has a wide range of pharmacological activities, including anti-inflammation, antioxidant, and anticancer activities [26]. WFA also showed potent liver-protective effects in nonalcoholic steatohepatitis [27] and acetaminophen-induced liver injury [28]. Interestingly, Sayed et al. reported that WFA could reverse bile duct ligation-induced liver fibrosis mainly by regulating extracellular matrix deposition [29]. However, whether regulation of oxidative stress involved in the antifibrogenic effect of WFA, and in particular, whether SIRT3 mediates the antioxidant stress effect of WFA in liver fibrosis has never been evaluated. Therefore, the present study is aimed at investigating the role of SIRT3 in WFA-induced antifibrotic and antioxidant effects. In addition, we employed carbon tetrachloride- (CCl_4_-) induced liver fibrosis in order to distinguish this study from a previous study.

## 2. Materials and Methods

### 2.1. Chemicals and Reagents

WFA (CAS. 5119-48-2, purity ≥ 99%) was purchased from Chengdu Push Bio-Technology Co., Ltd. (Chengdu, China). Primary antibodies against anti-rabbit *α*-SMA (14395-1-AP), *α*1(I) procollagen (14395-1-AP), fibronectin (15613-1-AP), collagen I (14695-1-AP), and GAPDH (13937-1-AP) were purchased from Proteintech Group, Inc. (Rosemont, IL, USA). Primary antibodies against SIRT3 (#5490) were purchased from Cell Signaling Technology (Danvers, MA, USA). The primers used in real-time PCR were from Nanjing Genscript Biotechnology Co., Ltd. (Nanjing, China). The Lipofectamine 2000 Transfection Reagent was from Suzhou Genepharma Co., Ltd. (Suzhou, China). The SIRT3 enzyme activity detection kit (JK50288.2) was purchased from Shanghai Baoman Biotechnology Co., Ltd. (Shanghai, China). Alanine transaminase (ALT) assay kit (C009-2-1), aspartate aminotransferase (AST) assay kit (C0010-2-1), alkaline phosphatase (ALP) assay kit (A059-1-1), hydroxyproline examination kit (A030-2-1), malondialdehyde (MDA) assay kit (A003-1-2), reduced glutathione (GSH) assay kit (A006-1-1), glutathione peroxidase (GPx) assay kit (A005-1-2), catalase (CAT) assay kit (A007-1-1), superoxide dismutase (SOD) typed assay kit (A001-2-2), glutathione reductase (GR) assay kit (A062-1-1), heme oxygenase 1 (HO-1, H246-1), total antioxidant capacity (T-AOC) assay kit (A015-1-2), and hyaluronic acid (HA) assay kit (H141) were purchased from Nanjing Jiancheng Bioengineering Institute (Nanjing, China). The laminin (LN) assay kit (SCA082Hu) was purchased from Beijing China Ocean Co., Ltd. The Type III Procollagen (PC-III) ELISA Kit (cby24659) was purchased from Nanjing Herb Source Bio-Technology Co., Ltd.

### 2.2. Animals and Experimental Procedures

All experimental procedures were approved by the institutional and local committee on the care and use of animals of Nantong University (Nantong, China), and all animals received humane care according to the National Institutes of Health (USA) guidelines. The detailed protocols were also approved by a specific committee in Nantong University (NTU-20181018).

For the liver fibrosis experiment, male 8-week-old C57/BL6 mice (20-22 g) were purchased from Shanghai SLAC Laboratory Animal Co. Ltd. They were housed in a temperature-controlled environment (22 ± 2°C) under standard 12 h light/dark conditions and received food and water *ad libitum*. A total of 32 male mice were randomly divided into four groups, i.e., the control group, the CCl_4_ group, the CCl_4_ + WFA (2.5 mg/kg) group, and the CCl_4_ + WFA (10 mg/kg) group, with eight mice per group. Liver fibrosis was induced by intraperitoneal (i.p.) injection of 20% CCl_4_ (diluted at 1 : 4 in olive oil (*v*/*v*)) (5 ml/kg body weight) twice a week for six weeks. Control mice were similarly treated with i.p. injection of the same volume of olive oil. All the treatment groups were injected intraperitoneally with WFA (dissolved in olive oil) at doses of 2.5 and 10 mg/kg once daily for six weeks. WFA was injected on the same day of CCl_4_ induction (for the dose of WFA, please refer to [29–31]).

For the liver fibrosis experiment, male 8-week-old adult 129S1/SvImJ (WT) and SIRT3 knockout (KO) mice (18-22 g) were provided by Professor Guoliang Meng (Nantong University). Mice were divided into six groups, i.e., WT + oil, WT + CCl_4_, WT + CCl_4_ + WFA (10 mg/kg), SIRT3 KO + oil, SIRT3 + CCl_4_, and SIRT3 + CCl_4_ + WFA (10 mg/kg), with seven mice per group. Liver fibrosis in groups WT + CCl_4_, WT CCl_4_ + WFA (10 mg/kg), SIRT3 + CCl_4_, and SIRT3 + CCl_4_ + WFA (10 mg/kg) was induced by intraperitoneal (i.p.) injection of 20% CCl_4_ (diluted in olive oil at 1 : 4 (*v*/*v*)) (5 ml/kg body weight) twice a week for 6 weeks [32]. Control mice were similarly treated with i.p. injection of the same volume of olive oil. All the treatment groups were injected intraperitoneally with WFA (dissolved in olive oil) at a dose of 10 mg/kg once daily for six weeks, WFA was injected on the same day of CCl_4_ induction. All mice were sacrificed 8 h after the last administration, and their venous blood and liver tissues were collected for further examination.

### 2.3. Liver Histopathology

Liver tissues were processed with formalin fixation, paraffin embedment, and sectioning and mounting on slides. For histopathological study, 5 *μ*m thick liver slices were prepared and stained with a hematoxylin and eosin staining kit (Nanjing best biotechnology Co., Ltd., China) according to the manufacturer's instructions. For Masson's trichrome staining, 5 *μ*m thick liver slices were prepared and stained with Masson's trichrome staining kit (Yeasen Biotech Co., Ltd., China) according to the manufacturer's instructions. For Picrosirius red staining, 5 *μ*m thick liver slices were prepared and stained with an enhanced Picrosirius red staining kit (Shanghai Zeye Biotechnology Co., Ltd., China) according to the manufacturer's instructions.

### 2.4. Biochemical Determinations

Levels of alanine aminotransferase (ALT), aspartate aminotransferase (AST), and alkaline phosphatase (ALP) in serum samples were evaluated using enzyme-linked immunosorbent assay methods according to the kit protocols (Nanjing Jiancheng Bioengineering Institute). The levels of serum procollagen-III (PC-III), hyaluronic acid (HA), and laminin (LN) were measured using ELISA kits according to the kit protocols, respectively. Experiments were performed in triplicate.

### 2.5. Immunofluorescence Analysis

For liver tissues, after deparaffinization, thin sections (5 *μ*m) of the liver tissues (liver lobules and portal area) were blocked with 1% bovine serum albumin, and then they were incubated with primary antibodies overnight at 4°C. After washing (×3) with PBS, sections were incubated with secondary antibodies at room temperature for 1 h. Sections incubated with secondary antibodies alone were used as negative controls. Sections were viewed in a single plane under an MRC 1024 laser confocal microscope (Bio-Rad Laboratories).

### 2.6. Real-Time PCR

Total RNA was extracted from mice liver samples or JS1 cells using the TRIzol Reagent (Biouniquer Technology Co., Ltd.) and then subjected to reverse transcription to cDNA using the kits provided by TaKaRa Biotechnology Co., Ltd. according to their protocol. An amplification kit was purchased from Bio-Rad Laboratories. GAPDH was used as the invariant control. Results were from triplicate experiments. Primers used for mouse reverse transcription-quantitative polymerase chain reaction in the present study are shown in Table 1.

### 2.7. SIRT3 Enzyme Activity Assay

SIRT3 enzyme activity in JS1 cells was detected using a SIRT3 enzyme activity detection kit (Shanghai Baoman Biotechnology Co., Ltd., Shanghai, China) and a colorimetric method according to the manufacturer's protocol; results were from triplicate experiments.

### 2.8. Cell Viability and Cytotoxicity Assays

The immortalized mouse hepatic stellate cell line JS1 was purchased from Nanjing Herb Source Bio-Technology Co., Ltd. The cells were cultured in DMEM with 10% fetal bovine serum and 1% antibiotics and maintained at 37°C in a humidified incubator with 5% CO_2_ and 95% air. The JS1 cells were seeded in 96-well plates; cultured in DMEM supplemented with 10% FBS for 24 h; and then treated with DMSO (0.02%, *w*/*v*), PDGF-BB (20 ng/ml), and the indicated concentrations of WFA for 24 h. WFA treatment was done at the same time with PDGF-BB. After treatment, 3-(4,5-dimethylthiazol-2-yl)-5-(3-carboxymethoxyphenyl)-2-(4-sulfophenyl)-2H-tetrazolium (MTS; Sigma-Aldrich, St. Louis, MO, USA) solution (5 mg/ml) was added (10 *μ*l/well), and the cells were further incubated for 3 h at 37°C. The spectrophotometric absorbance at 490 nm was measured by a SpectraMax™ microplate spectrophotometer (Molecular Devices, Sunnyvale, CA, USA). Five duplicates were set up for each group. For the cytotoxicity assay, lactate dehydrogenase (LDH) activity in culture medium was determined with a LDH release assay kit according to the manufacturer's protocol.

### 2.9. Cell Transfection

For cell transfection, the detailed procedure was conducted as previously described [33]. Briefly, SIRT3 siRNA or the negative control siRNA of 5 *μ*g was added to 100 *μ*l medium without serum and antibiotics and incubated at room temperature for 5 min. The Lipofectamine 2000 transfection reagent of 25 *μ*l was added to 75 *μ*l medium without serum and antibiotics and incubated at room temperature for 5 min. The above two solutions were mixed well at room temperature for 20 min, and about 200 *μ*l transfection complex was obtained. Then, the medium of 800 *μ*l without serum and antibiotics was added to the 200 *μ*l transfection complex and mixed well, and the transfection complex solution of 1000 *μ*l was obtained. JS1 cells were incubated with the transfection complex solution at 37°C for 8 h, and reincubated in complete medium at 37°C for an additional 16 h. The transfection efficiency was confirmed by Western blot analysis.

### 2.10. Western Blot Analyses

RIPA buffer supplemented with PMSF (Beyotime, Shanghai, China) was used for protein extraction from liver tissue and JS1 cells. The supernatants were used for the quantification of the total protein concentration by using a BCA Protein Assay Kit (Beyotime) according to the manufacturer's protocol. The protocols of the western blot analysis were based on our previously described protocols [34]. GAPDH was used as an invariant control for the target proteins. Representative blots are shown. Results were from triplicate experiments.

### 2.11. Liver Tissue or Cellular ROS Detection

The ROS content in the liver tissue or JS1 cells was measured using a DCFH-DA fluorescence probe by a reactive oxygen species detection kit (Beyotime). 10 *μ*l DCFH-DA working solution was mixed with 190 *μ*l liver homogenate supernatants and then incubated 30 min at 37°C in the dark. The fluorescence intensity was detected at an excitation wavelength of 500 nm and at an emission wavelength of 525 nm using a multimode reader (Varioskan Flash, Thermo Fisher Scientific). Results were from triplicate experiments.

### 2.12. MDA, GSH, and Antioxidant System Analyses

The contents of MDA and GSH and the activities of CAT, GR, HO-1, and SOD in liver tissue and JS1 cells were detected by using examination kits according to the protocols, respectively. The level of T-AOC was examined by using a T-AOC detection kit. Results were from triplicate experiments.

### 2.13. Statistical Analysis

All data were expressed as percentage and mean ± SD. Statistical analysis was performed using Student's *t*-test and one-way ANOVA by GraphPad Prism 5 for Windows. Values of *p* < 0.05 were considered to be statistically significant.

## 3. Results

### 3.1. WFA Inhibited PDGF-Induced Fibrotic Markers and Increased SIRT3 Expression in Activated JS1 Cells

**Figure 2:**
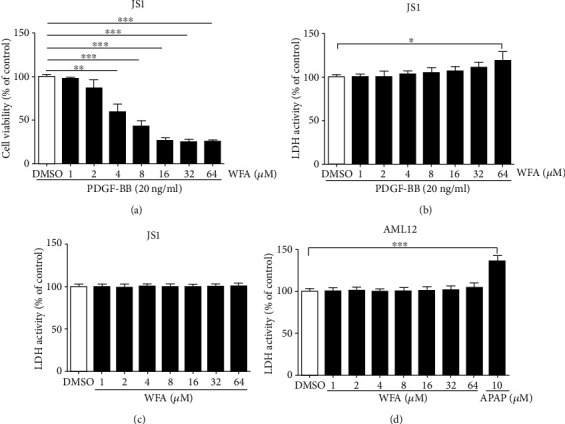
Effects of WFA on cell viability and toxicity of JS1 and AML12 cells. JS1 cells were treated with DMSO (0.02%, *w*/*v*), PDGF-BB (20 ng/ml), and the indicated concentrations of WFA for 24 h. (a) The effect of WFA on cell viability of activated JS1 cells by an MTS reagent. (b) The toxicity of WFA on activated JS1 cells was examined by a LDH activity assay kit. (c) The toxicity of WFA on inactivated JS1 cells was examined by a LDH activity assay kit. (d) AML12 cells were treated with DMSO (0.02%, *w*/*v*) and the indicated concentrations of APAP and WFA for 24 h. For the statistics of each panel in this figure, data are expressed as mean ± SD (*n* = 3). ^∗^*p* < 0.05, ^∗∗^*p* < 0.01, and ^∗∗∗^*p* < 0.001.

**Figure 3:**
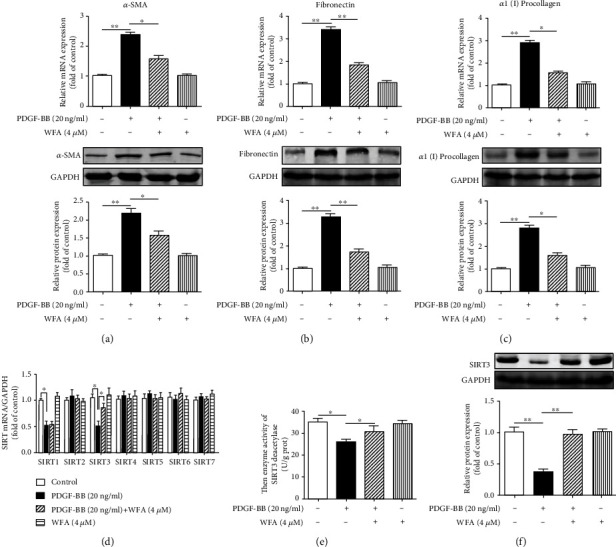
Effect of WFA on PDGF-BB-induced liver fibrosis and the expression of SIRT3 in activated JS1 cells. JS1 cells were treated with DMSO (0.02%, *w*/*v*), PDGF-BB (20 ng/ml), and the indicated concentrations of WFA for 24 h. (a–c) Quantification of *α*-SMA, fibronectin, and *α*1(I) procollagen expression in JS1 cells were carried out by real-time PCR and Western blot. (d) Quantification of SIR2 family (SIRT1-SIRT7) mRNA expression was assessed by real-time PCR. (e) The effect of WFA on the enzyme activity of SIRT3 deacetylase in activated JS1 cells. (f) Quantification of SIRT3 protein expression was assessed by Western blot in activated JS1 cells. For the statistics of each panel in this figure, data are expressed as mean ± SD (*n* = 3). ^∗^*p* < 0.05 and ^∗∗^*p* < 0.01.

We first evaluated the antifibrogenic effect of WFA in PDGF-BB-induced liver fibrosis in JS1 cells. WFA inhibited the cell viability of activated JS1 cells and showed a significant inhibitory effect at 4 *μ*M (Figure 2(a)). In addition, WFA showed toxicity on activated JS1 cells at 64 *μ*M (Figure 2(b)). We further studied the cytotoxicity effects of WFA on inactivated JS1 (without PDGF-BB treatment) and normal liver cell AML12 cells. WFA showed no obvious toxicity on inactivated JS1 (Figure 2(c)) and mice normal liver cell AML12 cells (Figure 2(d)) below 64 *μ*M, while acetaminophen (APAP) treatment notably induced toxicity on AML12 at 10 *μ*M (Figure 2(d)). Thus, given the above results, WFA (4 *μ*M) was chosen for the following experiments. WFA inhibited the mRNA and protein expression of *α*-SMA, fibronectin, and *α*1(I) procollagen compared to the PDGF-BB-treated group in activated JS1 cells (Figures 3(a)–3(c)). Noteworthy, WFA alone showed no obvious effect on the expression of the above proteins in JS1 cells (Figures 3(a)–3(c)). Of all the seven sirtuin members (SIRT1-SIRT7), only PDGF-BB treatment decreased the mRNA expression of SIRT1 and SIRT3. Interestingly, WFA increased SIRT3 but not SIRT1 mRNA expression in activated JS1 cells compared to the PDGF-BB-treated group (Figure 3(d)). We further examined the effect of WFA on the activity and protein expression of SIRT3 in activated JS1 cells. PDGF-BB treatment decreased the SIRT3 enzyme activity and protein expression, and WFA notably increased the enzyme activity and protein expression of SIRT3 in activated JS1 cells compared to the PDGF-BB-treated group (Figures 3(e)–3(f)). WFA alone had no obvious effect on the activity and protein expression of SIRT3 compared to the control group (Figures 3(e)–3(f)). Collectively, WFA inhibited PDGF-BB-induced fibrosis markers and increased SIRT3 expression in activated JS1 cells.

### 3.2. Depletion of SIRT3 Attenuated the Antifibrogenic and Antioxidant Effects of WFA in Activated JS1 Cells

**Figure 4:**
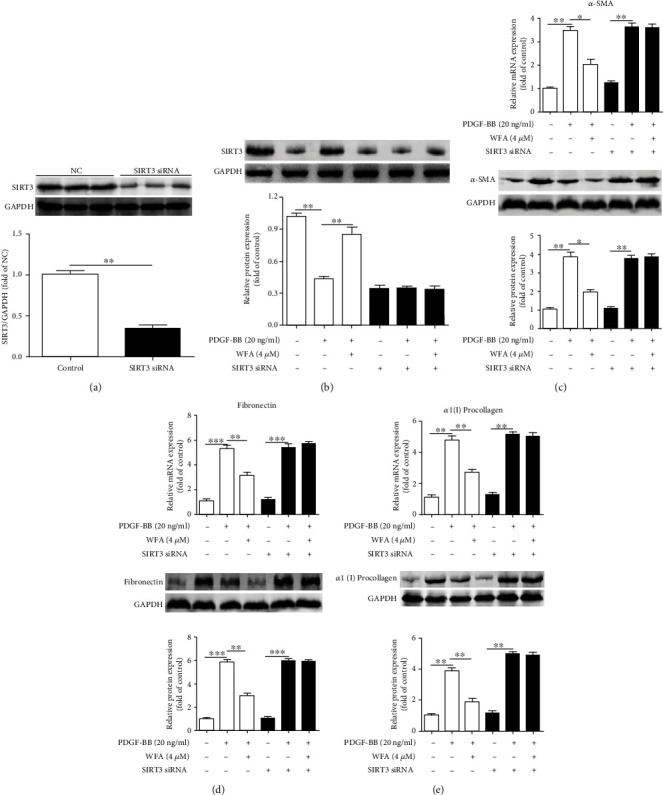
Effect of SIRT3 depletion on the antifibrogenic effect of WFA in activated JS1 cells. (a) After SIRT3 siRNA or NC siRNA was transfected into JS1 cells for 24 h, SIRT3 protein expression was measured with Western blots. (b) After SIRT3 siRNA or NC siRNA was transfected into JS1 cells for 24 h, cells were treated with DMSO (0.02%, *w*/*v*), PDGF-BB (20 ng/ml), and the indicated concentrations of WFA for 24 h. SIRT3 protein expression was measured by Western blots. (c–e) Quantification of *α*-SMA, fibronectin, and *α*1(I) procollagen expression in SIRT3-siRNA-treated or untreated JS1 cells (with NC siRNA transfection). For the statistics of each panel in this figure, data are expressed as mean ± SD (*n* = 3). ^∗^*p* < 0.05, ^∗∗^*p* < 0.01, and ^∗∗∗^*p* < 0.001.

**Figure 5:**
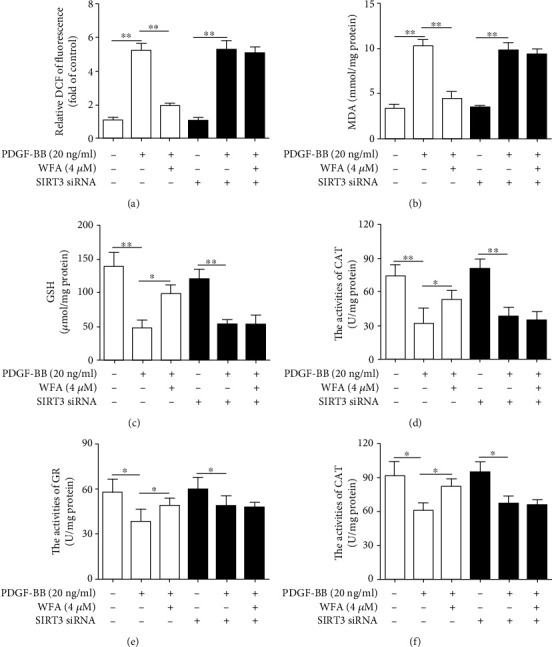
Effect of SIRT3 depletion on the antioxidant stress effect of WFA in activated JS1 cells. JS1 cells were treated with DMSO (0.02%, *w*/*v*), PDGF-BB (20 ng/ml), and the indicated concentrations of WFA for 24 h. (a) DCFH-DA staining was used to detect ROS production in the indicated groups. (b) The MDA assay kit was used to detect the MDA level in the indicated groups. (c) The GSH assay kit was used to detect the GSH level in the indicated groups. (d–f) The CAT, GR, and GPx assay kits were used to detect the activities of CAT, GR, and GPx in the indicated groups, respectively. For the statistics of each panel in this figure, data are expressed as mean ± SD (*n* = 3). ^∗^*p* < 0.05 and ^∗∗^*p* < 0.01.

The above data showed that WFA inhibited fibrosis markers and increased the SIRT3 expression in activated JS1 cells; however, whether SIRT3 enhancement is required for the WFA-induced inhibition of fibrosis markers remained to be determined. Thus, SIRT3 silencing was further used to elucidate the role of SIRT3 in the antifibrogenic effect of WFA. SIRT3 siRNA remarkably reduced the SIRT3 expression compared to the control siRNA (Figure 4(a)). WFA increased SIRT3 expression in PDGF-BB-induced JS1 cells (Figure 4(b)); however, SIRT3 silencing completely abolished the effects of WFA in activated JS1 cells (Figures 4(c)–4(e)). As oxidative stress is often concomitant with the development of liver fibrosis [35], we then studied the effect of SIRT3 silencing on oxidative stress regulated by WFA. PDGF-BB treatment increased the ROS production and MDA level; reduced the GSH level; and decreased the activities of CAT, GR, and GPx in activated JS1 cells (Figures 5(a)–5(f)). WFA significantly inhibited the ROS and MDA levels and notably increased the GSH level and the activities of CAT, GR, and GPx compared to the PDGF-BB-treated group in activated JS1 cells (Figures 5(a)–5(f)). However, SIRT3 silencing evidently attenuated the antioxidant effect of WFA in activated JS1 cells (Figures 5(a)–5(f)), suggesting that SIRT3 was essential for the antioxidant effect of WFA in liver fibrosis. Taken together, the depletion of SIRT3 attenuated the antifibrogenic and antioxidant effects of WFA in activated JS1 cells.

### 3.3. WFA Ameliorated the CCl_4_-Induced Liver Injury and Fibrosis in Mice

**Figure 6:**
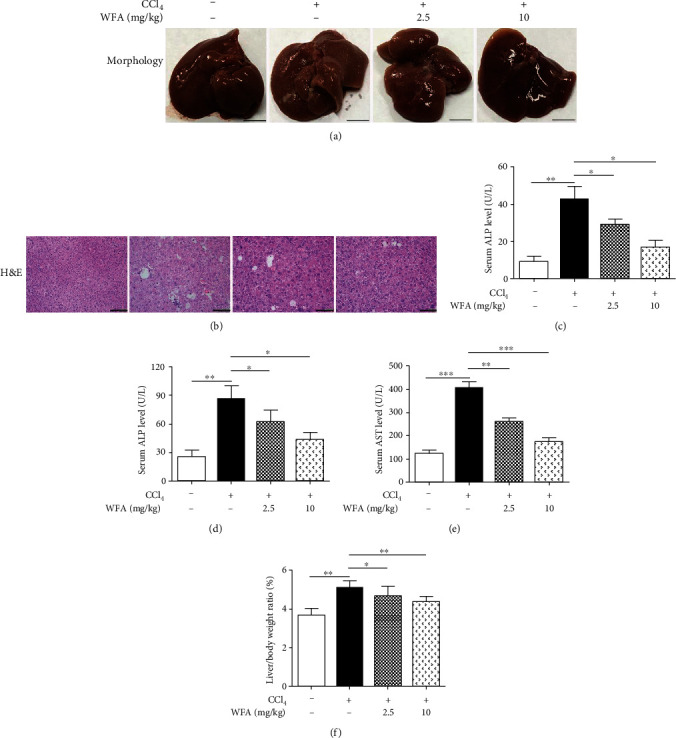
Effect of WFA on the CCl_4_-induced liver injury in mice. (a) Gross examination of livers in mice. Representative photographs are shown (scale bar: 1 cm). (b) Liver sections were stained with H&E for histological examination. Representative photographs are shown (scale bar: 100 *μ*m). (c–e) Determination of the serum levels of ALP, ALT, and AST. (f) The liver/body weight ratio (%). For the statistics of each panel in this figure, data are expressed as mean ± SD (*n* = 8/group). ^∗^*p* < 0.05, ^∗∗^*p* < 0.01, and ^∗∗∗^*p* < 0.001.

**Figure 7:**
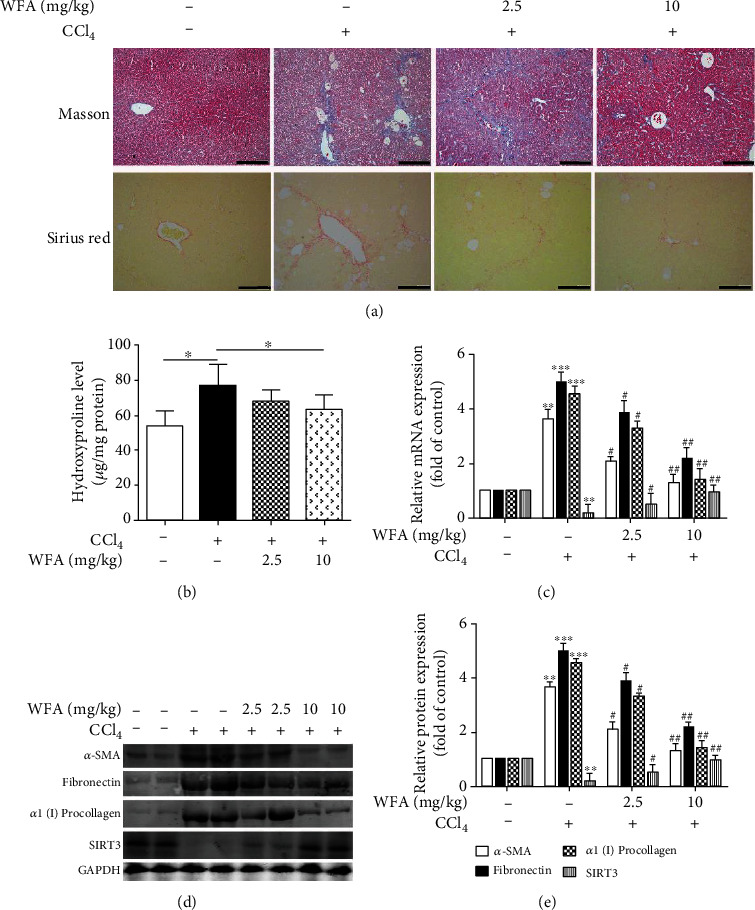
Effect of WFA on the CCl_4_-induced collagen production, liver fibrosis, and SIRT3 expression in mice. (a) Liver sections were stained with Masson's trichrome and picrosirius red reagents, and representative photographs are shown (scale bar: 100 *μ*m), respectively. (b) Measurement of hydroxyproline levels in liver homogenate, ^∗^*p* < 0.05. (c) Real-time PCR analyses of SIRT3, *α*-SMA, fibronectin, and *α*1(I) procollagen in liver tissues. GAPDH was used as the invariant control. (d) Western blot analyses of liver proteins with densitometry. Representative blots were from three independent experiments. (e) Quantified western blot results of liver proteins. For the statistics of each panel in this figure, data are expressed as mean ± SD (*n* = 3). ^∗∗^*p* < 0.01 and ^∗∗∗^*p* < 0.001 versus the control group; ^#^*p* < 0.05 and ^##^*p* < 0.01 versus the model (CCl_4_) group.

We further studied the antifibrogenic effect of WFA *in vivo*. WFA notably improved the morphological changes caused by CCl_4_ injection in livers of mice (Figure 6(a)). Furthermore, CCl_4_ administration caused hepatic steatosis, necrosis, and fibrotic septa in liver of mice, and WFA significantly improved the above pathological changes in livers of mice compared to the CCl_4_ group (Figure 6(b)). In addition, WFA inhibited the CCl_4_-induced high levels of ALP, ALT, and AST in serum of mice compared to the CCl_4_ group (Figures 6(c)–6(e)). Besides, WFA evidently decreased the CCl_4_-induced high ratio of liver/body weight in mice compared to the CCl_4_ group (Figure 6(f)). WFA suppressed the collagen deposition induced by CCl_4_ administration, as evidenced by the Masson's trichrome and Picrosirius red stainings (Figure 7(a)) together with the decreased hydroxyproline level (Figure 7(b)). Consistent with the *in vitro* results, WFA inhibited the mRNA and protein expression of *α*-SMA, fibronectin, and *α*1(I) procollagen and increased the SIRT3 expression in CCl_4_-injected livers of mice (Figures 7(c)–7(e)). Altogether, WFA attenuated the CCl_4_-induced liver injury and fibrosis in mice.

### 3.4. WFA Inhibited CCl_4_-Induced Oxidative Stress in Mice

**Figure 8:**
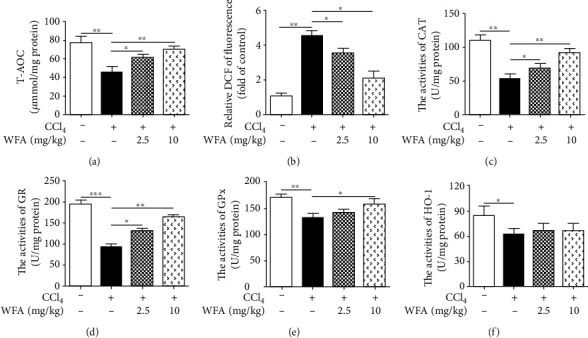
Effect of WFA on the CCl_4_-induced oxidative stress in mice. (a) A T-AOC assay kit was used to examine the T-AOC in livers of each group. (b) DCFH-DA staining was used to detect ROS production in livers of each group. (c–f) The CAT, GR, GPx, and HO-1 assay kits were used to detect the activities of CAT, GR, GPx, and HO-1 in the livers of each group, respectively. For the statistics of each panel in this figure, data are expressed as mean ± SD (*n* = 3). ^∗^*p* < 0.05, ^∗∗^*p* < 0.01, and ^∗∗∗^*p* < 0.001.

The antioxidant effect of WFA was further evaluated *in vivo*. CCl_4_ administration increased the ROS production and decreased the T-AOC level in livers of mice (Figures 8(a)–8(b)). Treatment with WFA significantly reduced the ROS level and increased the T-AOC level compared to the CCl_4_ group (Figures 8(a)–8(b)). We further studied the effect of WFA on the antioxidant enzyme system in livers of mice. In parallel with the *in vitro* results, CCl_4_ injection notably inhibited the enzyme activities of CAT, GR, GPx, and HO-1 in livers of mice (Figures 8(c)–8(f)). Treatment with WFA elevated the enzyme activities of CAT, GR, and GPx in a dose-dependent manner compared to the CCl_4_ group (Figures 8(c)–8(e)). However, WFA showed no obvious effect on HO-1 activity in livers of mice (Figure 8(f)). These data indicated that WFA suppressed the CCl_4_-induced oxidative stress in mice.

### 3.5. WFA Attenuated the CCl_4_-Induced Liver Injury and Fibrosis in WT Mice but Not in SIRT3 KO Mice

**Figure 9:**
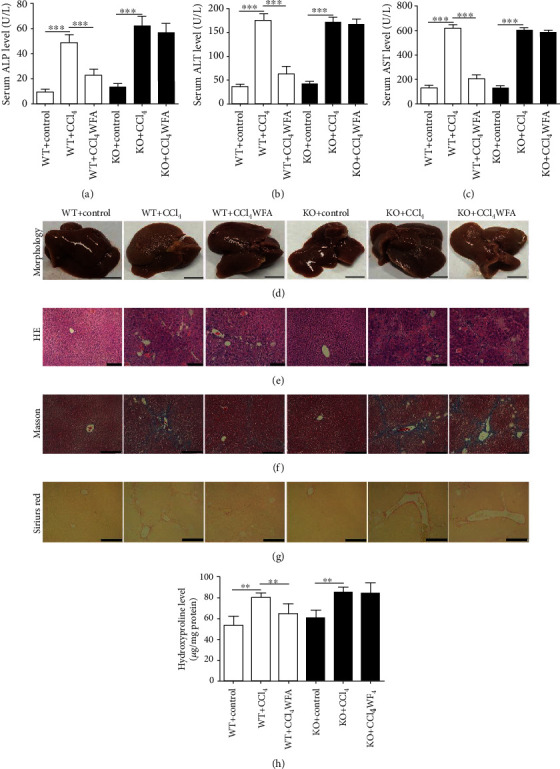
Effect of WFA on the CCl_4_-induced liver injury and collagen production in WT and SIRT3 KO mice. Male 129S1/SvImJ (WT) and SIRT3 KO mice were used to establish the model of liver fibrosis by intraperitoneal (i.p.) injections with 20% CCl_4_ (diluted in olive oil at 1 : 4 (*v*/*v*)) (5 ml/kg body weight) twice per week for 6 weeks, and all the treatment groups were injected i.p. with WFA (dissolved in olive oil) at a dose of 10 mg/kg for 6 weeks. (a–c) Serum levels of ALP, ALT, and AST were measured. (d) Gross examination of livers in WT and SIRT3 KO mice; representative photographs are shown (scale bar: 1 cm). (e) Liver sections were stained with H&E for histological examination. Representative photographs are shown (scale bar: 50 *μ*m). (f) Liver sections were stained with Masson's reagent for histological examination. Representative photographs are shown (scale bar: 100 *μ*m). (G) Liver sections were stained with the picrosirius red reagent for histological examination. (H) Measurement of hydroxyproline levels in liver homogenates of both WT and SIRT3 KO mice. Representative photographs are shown (scale bar: 100 *μ*m). For the statistics of each panel in this figure, data are expressed as mean ± SD (*n* = 3). ^∗∗^*p* < 0.01 and ^∗∗∗^*p* < 0.001.

**Figure 10:**
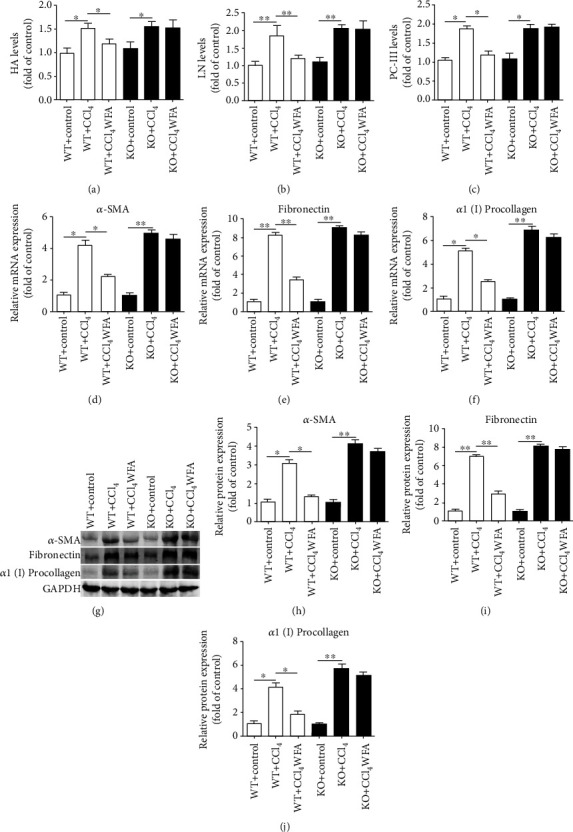
Effect of WFA on the CCl_4_-induced liver fibrosis in WT and SIRT3 KO mice. (a–c) The serum levels of HA, LN, and PC-III were examined in both WT and SIRT3 KO mice. (d–f) Real-time PCR analyses of *α*-SMA, fibronectin, and *α*1(I) procollagen in liver tissues. GAPDH was used as the invariant control. (g) Western blot analyses of liver proteins with densitometry. Representative blots were from three independent experiments. (h–j) Quantified western blot results of liver proteins. For the statistics of each panel in this figure, data are expressed as mean ± SD (*n* = 3). ^∗^*p* < 0.05 and ^∗∗^*p* < 0.01.

The above results suggested that WFA inhibited liver injury and fibrosis in mice; however, whether SIRT3 is required for the antifibrogenic effect of WFA in mice remains largely unknown. Therefore, SIRT3 KO mice were further used to elucidate the role of SIRT3 in WFA-induced liver fibrosis in mice. Compared with the control groups, CCl_4_ administration induced the elevated levels of ALP, ALT, and AST in serum of both WT and SIRT3 KO mice (Figures 9(a)–9(c)). Treatment with WFA reduced the CCl_4_-induced ALP, ALT, and AST levels in serum of WT mice but not in SIRT3 KO mice (Figures 9(a)–9(c)). CCl_4_-injection caused notable morphological and pathological changes in livers of both WT and SIRT3 KO mice (Figures 9(d)–9(e)). Compared to the model groups, WFA clearly improved the morphological and pathological changes in livers of WT mice but not in SIRT3 KO mice (Figures 9(d)–9(e)). Masson's trichrome and Picrosirius red stainings showed that WFA significantly reduced the CCl_4_-induced collagen deposition (Figures 9(f)–9(g)) in livers of WT mice but not in SIRT3 KO mice. A hydroxyproline level assay also obtained the same results (Figure 9(h)). HA, LN, and PC-III are three markers of the serologic index of liver fibrosis [36]. Our results showed that WFA reduced the serum levels of HA, LN, and PC-III in WT mice but not in SIRT3 KO mice (Figures 10(a)–10(c)). Real-time PCR analysis showed that CCl_4_-injection increased the mRNA expression of *α*-SMA, fibronectin, and *α*1(I) procollagen in livers of both WT and SIRT3 KO mice (Figures 10(d)–10(f)). Treatment with WFA inhibited the mRNA expression of the above proteins in WT mice but not in SIRT3 KO mice (Figures 10(d)–10(f)). Western blot analysis showed that CCl_4_-injection increased the expression of *α*-SMA, fibronectin, and *α*1(I) procollagen in livers of both WT and SIRT3 KO mice (Figures 10(g)–10(j)). Treatment with WFA inhibited the expression of the above proteins in WT mice but not in SIRT3 KO mice (Figures 10(g)–10(j)). Collectively, WFA ameliorated the CCl_4_-induced liver injury and fibrosis in WT mice but not in SIRT3 KO mice.

### 3.6. WFA Inhibited Oxidative Stress in Fibrotic Livers of WT Mice but Not in SIRT3 KO Mice

**Figure 11:**
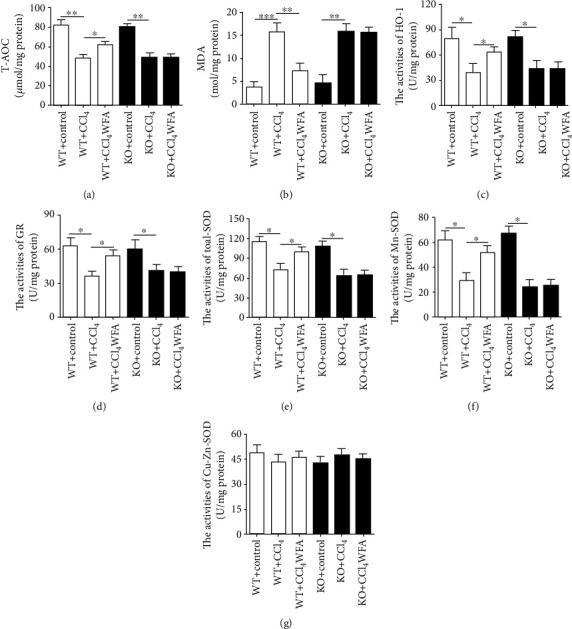
Effect of WFA on the oxidative stress in fibrotic livers of WT and SIRT3 KO mice. (a) A T-AOC assay kit was used to examine the T-AOC in livers of each group. (b) An MDA assay kit was used to examine the MDA level in livers of each group. (c–g) The enzyme activities of HO-1, GR, total SOD, Mn-SOD, and Cu-Zn/SOD were measured in livers of each group. For the statistics of each panel in this figure, data are expressed as mean ± SD (*n* = 3). ^∗^*p* < 0.05, ^∗∗^*p* < 0.01, and ^∗∗∗^*p* < 0.001.

To determine whether the antifibrogenic effect of WFA in mice was associated with the SIRT3-dependent oxidative stress, the oxidative stress level was examined in fibrotic livers of WT and SIRT3 KO mice. CCl_4_ injection reduced the level of T-AOC and increased the level of MDA in livers of both WT and SIRT3 KO mice (Figures 11(a)–11(b)). Treatment with WFA clearly elevated the T-AOC level but reduced the MDA level in livers of WT mice but not in SIRT3 KO mice (Figures 11(a)–11(b)). Compared to the model group, examination of the enzyme activities of the antioxidant showed that WFA elevated the activities of HO-1, GR, total SOD, and Mn-SOD in livers of WT mice but not in SIRT3 KO mice (Figures 11(c)–11(f)). However, WFA showed no detectable effect on the enzyme activity of Cu-Zn-SOD both in livers of WT mice and SIRT3 KO mice (Figure 11(g)). Altogether, SIRT3 was essential for the antioxidant effect of WFA in CCl_4_-induced liver fibrosis in mice.

## 4. Discussion

Accumulating evidence [37, 38], including ours [39], has highlighted that inhibition of oxidative stress helps to control the progression of liver fibrosis. In the present study, we proved that WFA inhibited the PDGF-BB-induced *in vitro* fibrotic model and CCl_4_-induced liver fibrosis mainly by the inhibition of oxidative stress. Examination of several factors associated with oxidative stress demonstrated the decreased levels of ROS and MDA and the increased level of GSH induced by WFA. Besides, the antioxidant system was found to be involved in the antifibrogenic effect of WFA, and the activities of several antioxidant enzymes were notably elevated by WFA. In particular, of all the seven sirtuins, only SIRT3 was identified to participate in the antifibrogenic effect of WFA. WFA promoted the SIRT3 expression, while depletion or knockout of SIRT3 ameliorated the antioxidant effect of WFA in liver fibrosis. To our knowledge, no reports can be seen evaluating the antifibrogenic effect by regulating the SIRT3-dependent inhibition of oxidative stress.

Despite the increasing understanding towards the pathogenesis of liver fibrosis, there are still no effective drugs or therapeutic methods in clinical trials. Therefore, identifying antifibrogenic agents that are innocuous is urgently needed [40]. Natural herbal plant-derived products have drawn worldwide attention due to their extensive biological activities. WFA is a natural steroidal lactone, rejuvenating tonifier, and immunomodulator obtained from *Withania somnifera* [41]. WFA is proven to have a notable hepatoprotective effect against several liver-related diseases. Patel et al. reported that WFA prevented and improved liver injury in nonalcoholic steatohepatitis [27], even in high-fat-diet-treated leptin-deficient ob/ob mice, indicating that the hepatoprotective effect of WFA was in a leptin-independent manner in nonalcoholic steatohepatitis. Palliyaguru et al. found that WFA inhibited acetaminophen- (APAP-) induced hepatic toxicity in WT mice but not in Nrf2-disrupted mice, suggesting that the hepatoprotective effect of WFA was in an Nrf2-dependent manner in APAP-induced hepatic toxicity [42]. Interestingly, WFA inhibited bile duct ligation-induced liver fibrosis in mice mainly by modulating extracellular matrix deposition, and it was further shown that lysyl oxidase like 2 (LOXL2), snail, vimentin, and NF-*κ*B signaling were involved in the hepatoprotective effect of WFA against liver fibrosis [29]. In addition, WFA inhibited the vimentin intermediate filament (VimIF) assembly and induced autophagy in the idiopathic pulmonary fibrosis fibroblast, and further demonstrated that WFA-induced VimIF disruption could increase collagen type I turnover in autophagosomes in IPF fibroblasts, and then exert its antifibrotic effects [43]. Consistent with the previous studies, our data showed that WFA inhibited the PDGF-BB-induced fibrotic markers and attenuated the CCl_4_-induced liver fibrosis mainly by inhibiting oxidative stress in fibrotic livers and activated HSCs, highlighting that the antioxidative stress effect may contribute to the antifibrogenic effect of WFA. Noteworthy, WFA alone had no obvious effect on the expression of markers of liver fibrosis, showing that WFA exerted its hepatoprotective effect only under pathological conditions such as liver fibrosis. Another point worth noting is that the bioavailability of WFA is poor; pharmacokinetic studies found that after oral (intragastric, i.g.) administration in rats, the distribution of WA in the various tissues was in the following order: stomach > heart > lung > kidney > small intestine > spleen > liver. In addition to the stomach, blood-rich tissues including the heart, lung, and kidney had a high WFA content, suggesting that the blood flow could influence the distribution of WFA [44]. Moreover, the pharmacokinetic properties of withanolides (such as WFA and withanolide A) were characterized with rapid oral absorption following oral administration of *W. somnifera* root aqueous extract (WSE) [45]. According to the PK data, we can design and develop newer rationale oral preparations in the future, such as oral liquid, oral solution, oral suspension, and oral emulsion. Also, we can study herb-drug interactions potentially influencing absorption and metabolism during concurrent administration with other prescription drugs.

Previous reports suggested that SIRT3 was involved in the occurrence and development of liver-associated diseases including liver fibrosis [46, 47]. Li et al. found that a chronic high-fat diet caused the downregulation of SIRT3 in liver tissue concomitant with the impaired liver function and inflammatory response. Moreover, SIRT3 overexpression protected hepatic function, attenuated liver fibrosis, alleviated the inflammatory response, and prevented hepatocyte apoptosis, making SIRT3 a potential therapeutic target for the treatment of nonalcoholic fatty liver disease [48]. Wang et al. reported that *γ*-mangostin attenuated liver fibrosis by inhibiting NAD(P)H oxidase activity through SIRT3 enhancement, resulting in reduced intracellular oxidative stress [49]. Besides, our previous study showed that celastrol increased SIRT3 promoter activity and SIRT3 expression both in fibrotic liver and in activated HSCs. SIRT3 siRNA attenuated the anti-inflammation effect of celastrol in liver fibrosis [15]. In line with the previous studies, this study found that WFA is a potent inducer of SIRT3 and increased the SIRT3 expression both in a PDGF-BB-induced *in vitro* fibrotic model and a CCl_4_-induced *in vivo* liver fibrosis model, and SIRT3 siRNA or SIRT3 knockout attenuated the antifibrogenic effect of WFA mainly by the inhibition of oxidative stress, thus further suggesting that SIRT3 is a promising therapeutic target for the treatment of liver-related diseases. Noteworthy, of all the seven sirtuins, only SIRT1 and SIRT3 could be downregulated by PDGF-BB, which indicates that both SIRT1 and SIRT3 are involved in the PDGF-BB-induced *in vitro* fibrotic model; however, only SIRT3 can be enhanced by WFA. Actually, previous studies have shown that SIRT1 played an essential role in guiding the transition of HSC phenotypes, and SIRT1 activation halted, whereas SIRT1 inhibition promoted, HSC transdifferentiation into myofibroblasts. Besides, liver fibrosis was exacerbated in mice with HSC-specific deletion of SIRT1 [50]. In addition, SIRT1 downregulation is concomitant with HDAC4 upregulation. HDAC4 was recruited to the SIRT1 promoter during HSC activation and removed acetylated histones H3 and H4 from the SIRT1 promoter leading to SIRT1 transrepression [51]. The above studies indicated that SIRT1 exerted an important role during the development of liver fibrosis. However, WFA had no obvious effect on SIRT1 expression but notably increased the SIRT3 expression, suggesting that SIRT3 but not SIRT1 contributed to the preventive effect of WFA on liver fibrosis. Nonetheless, how WFA regulates SIRT3 and the potential underlying mechanisms remain to be determined. Next, whether WFA binds to SIRT3 directly or by indirect regulation will be studied using molecular docking and coimmunoprecipitation.

SIRT3 is a deacetylase which is critical for antioxidant protection, cell longevity, and aging [52]. SIRT3 mediates oxidative stress mainly by regulating the activities of mitochondrial antioxidant enzymes such as CAT, GR, GPx, and SOD [53]. Dikalova et al. reported that SIRT3 depletion led to SOD2 hyperacetylation and caused SOD2 inactivation, then increased the mitochondrial oxidative stress in the angiotensin II model of hypertension. Furthermore, the decreased SIRT3 expression concomitant with the increased SOD2 acetylation could also be observed in hypertensive patients [54]. Han et al. found that phloretin effectively attenuated palmitic acid- (PA-) induced oxidative stress by promoting the expression of SIRT3 in endothelial cells, then the increased SIRT3 expression led to the decreased lysine acetylation of Mn-SOD and the restored of Mn-SOD activity, exerting its antioxidant effect [55]. Zhang et al. found that SIRT3 could protect aged human mesenchymal stem cells (hMSCs) against oxidative stress by positively regulating antioxidant enzymes Mn-SOD and CAT. SIRT3 overexpression increased the antioxidant capacity of hMSCs, while SIRT3 silence decreased the antioxidant capacity [56]. In the present study, SIRT3 depletion or SIRT3 KO clearly attenuated the promoting effect of WFA on the activities of CAT, GR, GPx, HO-1, and SOD both in activated HSCs and in fibrotic livers, which further suggested that SIRT3 mediates the oxidative stress mainly through the regulation of the activities of a mitochondrial antioxidant enzyme.

**Figure 12:**
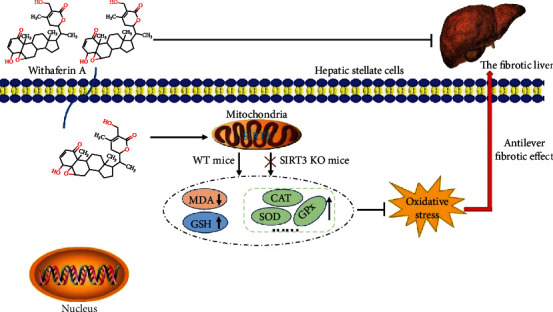
Schema of the underlying mechanism of withaferin A inhibition of liver fibrosis. Withaferin A enters into hepatic stellate cells and activates SIRT3 (mainly located in mitochondria), then SIRT3 activation causes the decreased MDA level and increased GSH level. In addition, SIRT3 activation also increases the activities of antioxidant enzymes such as CAT, SOD, and GPx leading to the inhibition of oxidative stress, thus exerting the antiliver fibrotic effect. However, the antioxidative stress and antifibrotic effects of withaferin A can only be observed in WT mice but not in SIRT3 KO mice. Overall, withaferin A inhibits liver fibrosis by suppressing oxidative stress in a SIRT3-dependent manner.

In summary, we demonstrated that WFA attenuated the liver fibrosis by inhibiting oxidative stress in a SIRT3-dependent manner (Figure 12). These data not only confirmed and extended the prior knowledge on the effect of WFA on liver fibrosis but also provided novel insights into the mechanisms involved. WFA is a promising compound for the prevention and treatment of liver fibrosis.

## Figures and Tables

**Table 1:** The sequences of the primers for real-time PCR.

Gene	Forward primers	Reverse primers
*α*-SMA	5′-CCACCATCTGCCTGAAATCC-3′	5′-GCTTCTTGTCCAGCCTCCTC-3′
Fibronectin	5′-ACACGGTTTCCCATTACGCC-3′	5′-GGTCTTCCCATCGTCATAGCAC-3′
*α*1(I) Procollagen	5′-GCTCCTCTTAGGGGCCACT-3′	5′-CCACGTCTCACCATTGGGG-3′
SIRT1	5′-AGTTCCAGCCGTCTCTGTGT-3′	5′-GATCCTTTGGATTCCTGCAA-3′
SIRT2	5′-TAGACACGCTGGAACGAGTG-3′	5′-TCTCTTTCATCCAGCCCATC-3′
SIRT3	5′-CAGCTACATGCACGGTCTGT-3′	5′-ACACAATGTCGGGTTTCACA-3′
SIRT4	5′-CATCCAGCACATTGATTTCG-3′	5′-CCAGTCTCTCCCAGTTGCTC-3′
SIRT5	5′-CCATCACCCAGAACATTGACG-3′	5′-ACAGTGCCACACGAGGTACA-3′
SIRT6	5′-GTGGATGAGGTGATGTGCAG-3′	5′-TCAGCCTTGAGTGCTACTGG-3′
SIRT7	5′-GACTGAGCGTACTGCCCTTC-3′	5′-TTCAGAGGCTGCCCTAATGT-3′
GAPDH	5′-TGAACGGGAAGCTCACTGG-3′	5′-GCTTCACCACCTTCTTGATGTC-3′

## Data Availability

The data used to support the findings of this study are available from the corresponding authors upon request.
